# Economic evaluation of AI-based oral disease screening: a systematic review

**DOI:** 10.3389/fmedt.2026.1792401

**Published:** 2026-06-25

**Authors:** Rakesh Kumar Sahoo, Abhinav Sinha, Sayani Satpathi, Gunjan Kumar, Krushna Chandra Sahoo, Debdutta Bhattacharya, Bhuputra Panda, Sanghamitra Pati

**Affiliations:** 1 School of Public Health, Kalinga Institute of Industrial Technology (KIIT), Deemed to be University, Bhubaneswar, Odisha, India; 2 Health Technology Assessment Regional Resource Hub (HTAIn), ICMR -Regional Medical Research Centre, Bhubaneswar, Odisha, India; 3 Department of Evidence Synthesis, South Asian Institute of Health Promotion, Bhubaneswar, Odisha, India; 4 Department of Public Health, ICMR -Regional Medical Research Centre, Bhubaneswar, India; 5 Department of Public Health Dentistry, Kalinga Institute of Dental Sciences (KIDS), Kalinga Institute of Industrial Technology (KIIT) Deemed to Be University, Bhubaneswar, India; 6 Department of Health Research, Ministry of Health & Family Welfare, Health Technology Assessment in India (HTAIn), Government of India, New Delhi, India

**Keywords:** artificial intelligence, deep learning, health economic analysis, incremental cost-effectiveness ratio, machine learning, quality adjusted life years

## Abstract

**Objectives:**

Artificial intelligence (AI) has made a significant contribution to dentistry, particularly in improving screening and diagnostic accuracy. This systematic review assessed the cost-effectiveness and broader economic implications of incorporating AI into oral disease screening.

**Methods:**

We included studies that conducted economic analyses and/or cost-effectiveness evaluations of AI-driven tools compared with conventional techniques for detecting oral diseases. We searched Cochrane Library, CINAHL, Embase, PubMed, Scopus, and Web of Science. The search strategy combined terms related to economic evaluation, artificial intelligence, and oral diseases. The review followed the Preferred Reporting Items for Systematic Reviews and Meta-Analyses (PRISMA) guidelines and was registered in PROSPERO (CRD42024548039). Articles published up to January 12, 2025, were included.

**Results:**

Of 334 records identified, four studies met the inclusion criteria - all these studies dealt with dental economic evaluations, and were conducted in the German dental care settings. All four evaluated AI-assisted detection of dental or proximal caries on bitewing radiographs. Across studies, AI generally improved diagnostic performance, particularly sensitivity. In one modelling study, a U-Net–based system achieved higher accuracy than dentists (0.80 vs. 0.71). Three model-based economic evaluations reported lower costs and greater tooth-retention outcomes with AI than with conventional assessment. Costs ranged from €298 to €378 for AI and from €322 to €419 for conventional care. Tooth retention ranged from 62.4 to 64.0 years with AI and from 60.4 to 62.0 years with conventional care. In contrast, the randomized trial found that AI-supported assessment increased sensitivity but was associated with more invasive treatment decisions. This resulted in similar costs (€330 vs. €330) and identical mean tooth retention (49 years) compared with standard assessment. Overall, the evidence suggests a potential economic benefit of AI in caries detection, but uncertainty remains under real-world treatment conditions.

**Conclusions:**

AI-assisted screening may offer economic value in dental caries detection, particularly when improved lesion detection is linked to appropriate downstream management. These findings are relevant for clinicians, payers, and health-system decision-makers considering the integration of AI into diagnostic pathways. Future multicentre, real-world economic evaluations are needed to guide reimbursement, implementation, and equitable adoption across diverse settings.

**Systematic Review Registration:**

https://www.crd.york.ac.uk/PROSPERO/view/CRD42024548039, PROSPERO: CRD42024548039.

## 1 Introduction

Digital technologies have transformed dentistry by introducing innovative care models that expand the scope and quality of oral healthcare (1–3). Among these technologies, artificial intelligence (AI) has emerged as a promising tool in dental radiology. AI methods, including deep learning and convolutional neural networks (CNNs), can support automated image assessment and may reduce diagnostic errors by up to 40% (3–6). By improving the speed and consistency of radiographic interpretation, AI can assist clinicians in disease detection and allow greater focus on complex cases (7, 8).

**Table 1:** Characteristics of the included studies.

Author, Year	Country	Disease	Method	AI Model Performance Vs Dentists	Dentists with AI (Costs in €)	Dentists without AI (Costs in €)	Incremental Costs in Euro	Direction of Incremental Costs	Incremental Cost-Effectiveness Ratios (ICER)	Costs per QALY Gained	Perspective of Evaluation	Whether Cost-Effective	Outcomes
F. Schwendicke et al., (3)	Germany	Dental Caries	CEA^a^	NA	€378 (Range: €284–€499)	€419 (Range: €270–€593)	-€ 41	Cheaper in the AI Group	NA	Not directly mentioned; Outcome measured in tooth retention years: 62.8 (AI) vs. 60.4 (No AI)	Mixed public-private payer	Positive	AI reduced costs and improved tooth retention, demonstrating cost-effectiveness when datasets were expanded for accuracy.
F. Schwendicke et al., (15)	Germany	Dental Caries	CEA	NA	€330 (Range: €250–€409)	€330 (Range: €248–€410)	-€ 8.9/year	Same in both groups	-€ 8.9/year	Not explicitly provided; effectiveness in tooth retention years: 49 years (both groups)	Mixed public-private payer perspective	Mixed (Negative in Base Case, Positive in Sensitivity Analyses)	This trial found AI-supported caries detection improved sensitivity but led to invasive treatments, with mixed cost-effectiveness depending on conditions like reduced costs or non-AI accuracy.
Jesus Gomez Rossi et al., (16)	Germany, United States, and Brazil	Dental Caries	CEA	NA	€320 (95% CI, €299-€341)	€342 (95% CI, €318-€368)	-€ 22	Cheaper in the AI Group	-€15.01 per tooth retention year	Not explicitly provided	Payer perspective	Positive	AI improved caries diagnosis, saving costs and increasing tooth retention (62.4 vs. 60.9 years), highlighting its potential to enhance dental care and cost-effectiveness.
F. Schwendicke et al., (17)	Germany	Proximal Caries	CEA	Sensitivity: 0.75 (AI) vs. 0.36 (Dentists); Specificity: 0.83 (AI) vs. 0.91 (Dentists)	€298 (Range: €244–€367)	€322 (Range: €257–€394)	-€ 24	Cheaper in the AI Group	-€13.9 per tooth retention year	Not explicitly provided; effectiveness measured as tooth retention: 64 years (AI) vs. 62 years (No AI)	Mixed public-private payer perspective	Positive	AI-assisted caries detection improved sensitivity, reduced costs, and increased tooth retention, showing positive cost-effectiveness dependent on non-invasive management, confirmed by sensitivity analyses.

Diagnostic performance data are presented only where direct comparative AI-vs.-dentist metrics were reported in the included studies. Cost uncertainty estimates (range or 95% CI) are presented as originally reported in the source articles.

a CEA, cost-effectiveness analysis; NA, not available .

Despite these benefits, the transition to AI-integrated practice is hindered by factors such as data sharing and privacy concerns, lack of algorithm transparency, and the need for standardized, interoperable data (9). Beyond these technical and ethical challenges, the economic implications of AI in dentistry remain under-evaluated. While AI holds potential for streamlining treatment planning and administrative efficiency, current research provides limited data to guide evidence-based policymaking or assess the true return on investment for dental practices (10, 11).

Health economic analysis (HEA) provides a structured framework to determine whether the clinical benefits of an intervention justify its costs (12–14). In general healthcare, economic evaluations often rely on metrics such as life expectancy or mortality reduction. However, these measures are less applicable to dentistry. Instead, dental studies commonly use outcomes such as tooth retention, lesion progression, and treatment costs (15–18). Commonly employed methods include cost-utility analysis, which uses quality-adjusted life years (QALYs) or “tooth-years saved” to capture both the quality and duration of oral health outcomes. Cost-minimization analysis compares the least expensive option among interventions with equivalent outcomes. Cost–benefit analysis converts clinical outcomes into monetary values (12–14, 18).

This systematic review addresses the current gap in knowledge by evaluating the economic impact of AI-based screening for oral diseases. It aims to provide insights into the financial feasibility and potential value of AI in oral healthcare while identifying existing gaps and opportunities to guide future research, policy development, and resource allocation in oral healthcare.

## 2 Methods

This systematic review was registered with the International Prospective Register of Systematic Reviews (PROSPERO; Registration ID: CRD42024548039) and conducted in accordance with the Preferred Reporting Items for Systematic Reviews and Meta-Analyses (PRISMA) guidelines (19). We considered ’systematic review’ as the most appropriate methodology for this study because the review aimed to identify, critically appraise, and synthesize economic evidence on AI-based screening for oral diseases. Moreover, systematic reviews of economic evaluation are recommended for summarizing cost-effectiveness evidence across interventions, settings, and modelling approaches (20). This methodology enabled structured comparison of cost-effectiveness outcomes across different models and settings; it also promoted methodological transparency and helped reduce selection bias, leading to a better reliability on the findings.

### 2.1 Data sources, search strategy, and inclusion criteria

We conducted a comprehensive search across six databases: CINAHL, Cochrane Library, Embase, PubMed, Scopus, and Web of Science. These databases were selected for their extensive coverage of healthcare, biomedical research, and economic evaluations. The search included articles published up to January 12, 2025, without imposing a start date limit, to capture all potentially relevant studies. Only English-language articles were included to ensure consistent data interpretation.

Our search strategy combined three main concepts: Economic Evaluation, Artificial Intelligence, and Oral Diseases. We used specific keywords and Medical Subject Headings (MeSH) terms, including combinations of “Oral diseases,” “Oral lesions,” or “Oral Health” with “Deep Learning,” “Artificial Intelligence,” “Machine Learning,” or “Neural Network,” and economic terms such as “Cost-Effectiveness Analysis,” “Economic Evaluation,” “Cost-Utility Analysis,” “Marginal Analysis,” “Cost-Benefit Analysis,” and “Health Care Economics.” The Boolean operator ‘OR’ was used to combine MeSH terms and keywords of the same concept, while ‘AND’ was used to combine the three concepts. Database-specific controlled vocabulary, such as MeSH terms in PubMed and Emtree terms in Embase, along with the associated keywords, were used in combination. For example, in PubMed, MeSH terms such as “Artificial Intelligence,” “Oral Health,” and “Cost-Benefit Analysis” were combined with keyword searches using truncation and field tags. The search strategies were tailored to each database (Supplementary Table 1).

We included original studies evaluating the economic efficiency of AI-based screening or diagnostic methods for oral diseases compared with conventional approaches. Conference abstracts, non-original studies (e.g., reviews and editorials), studies lacking sufficient economic data, and those not focused on AI-based tools were excluded. Grey literature (e.g., HTA reports and WHO databases) was not systematically searched, as the review prioritized peer-reviewed evidence to ensure methodological consistency and comparability. This may have limited the inclusion of relevant unpublished or policy-level evidence and is acknowledged as a limitation. Nevertheless, the inclusion criteria supported the synthesis of high-quality evidence aligned with the study objectives.

### 2.2 Data extraction and synthesis

We selected articles that applied health economic analysis (HEA) to compare AI-based vs. traditional screening methods for oral diseases across different populations. Records were retrieved in RIS format, deduplicated using EndNote, and screened using Rayyan. Two reviewers (RKS, AS) independently screened titles and abstracts, excluding irrelevant studies by mutual agreement. For uncertain cases, a third reviewer (SS) provided additional evaluation. Full-text eligibility was independently assessed by two reviewers (RKS, AS) based on predefined criteria, with disagreements resolved by discussion or by a senior reviewer (KCS) if needed.

Data extraction was performed using a pre-piloted form. The form captured author details, publication year, disease type, analysis method, AI model performance, incremental costs, incremental quality-adjusted life years (QALYs), incremental cost-utility ratios (ICURs), incremental cost-effectiveness ratios (ICERs), and cost-effectiveness outcomes. Two reviewers (RKS and AS) independently extracted the data. Discrepancies were resolved through discussion with other reviewers (BP and SP). For studies with missing or incomplete data, we contacted the authors directly.

We analysed four key cost-effectiveness indicators: (1) Incremental Cost (additional expense relative to standard care); (2) Incremental QALYs (benefit in quality and duration of life); (3) ICUR (cost per unit of utility gained); and (4) ICER (relative costs and effects of two interventions).

### 2.3 Quality assessment

Two authors (RKS and GK) independently assessed risk of bias using the Joanna Briggs Institute (JBI) Critical Appraisal Checklist for Economic Evaluations. This tool was selected because it provides comprehensive criteria for assessing economic evaluations. The checklist includes eleven criteria rated as ‘yes,’ ‘no,’ ‘unclear,’ or ‘not applicable (20). Discrepancies were resolved through discussion. If disagreement persisted, a senior reviewer (KCS) was consulted. We also assessed reporting completeness using the CHEERS 2022 checklist (12). CHEERS 2022 is a reporting guideline rather than a risk-of-bias tool. Therefore, it was used to complement, rather than replace, the JBI-based appraisal. Studies were rated as reported, partially reported, not reported, or not applicable across the 28 CHEERS 2022 items. The results are presented in Supplementary Table 2.

## 3 Results

**Figure 1:**
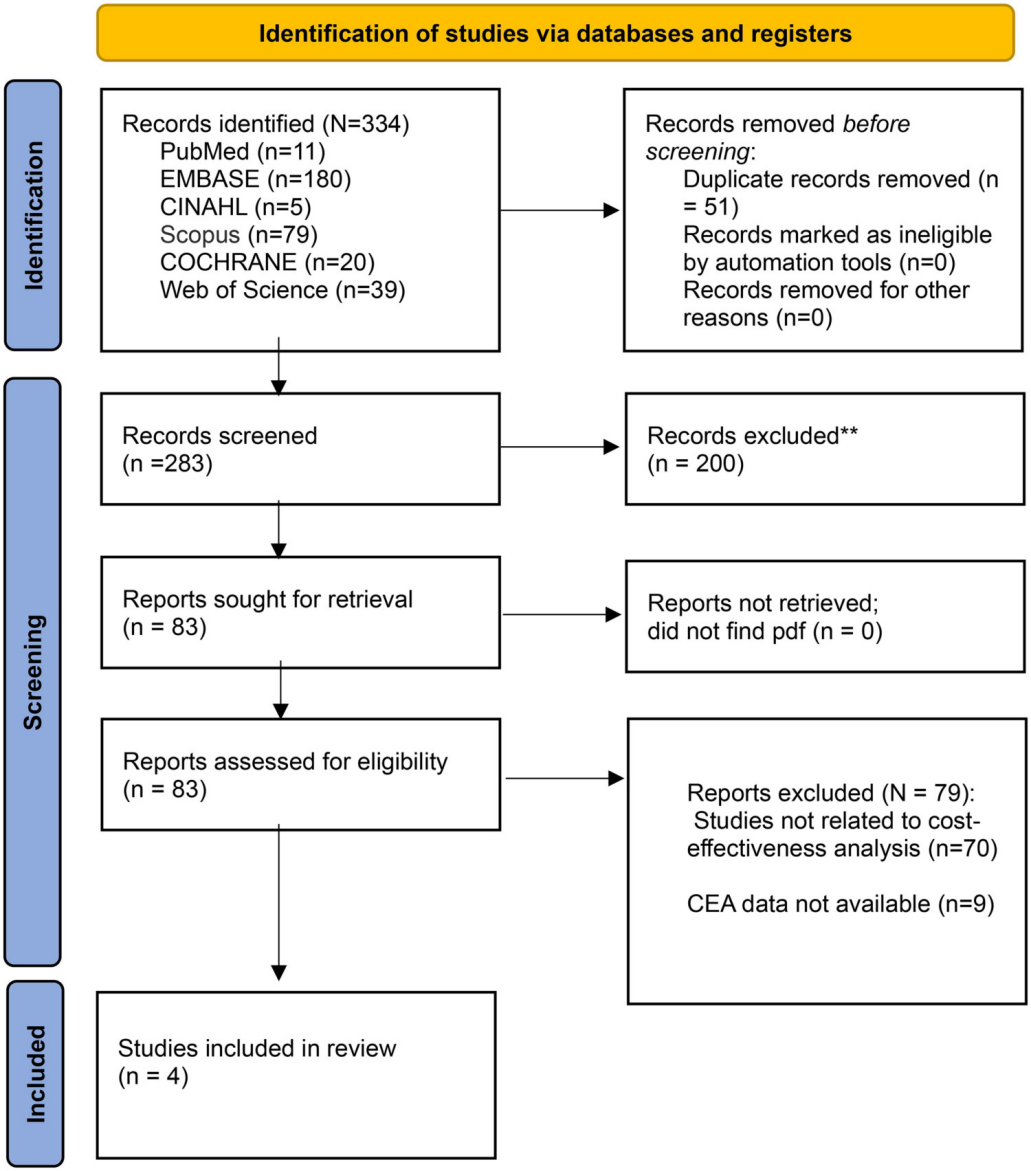
PRISMA flow diagram.

The study retrieved a total of 334 records, out of which 51 duplicates were removed, selecting 283 for primary screening based on title and abstract. After thorough screening, 83 articles were identified as potentially eligible for full-text review. Of these, four articles (3, 15–17) met the eligibility criteria and were included in the review. Figure 1 illustrates the PRISMA flow diagram.

### 3.1 Study characteristics

The characteristics of the included studies are summarized in Table 1. The four included studies were comparable in their overall clinical focus but differed in study design and analytical purpose. Three studies were model-based economic evaluations (3, 16, 17). One study was a randomized clustered cross-over trial that generated primary diagnostic and treatment-decision data and incorporated these findings into a cost-effectiveness model (15). Three studies were conducted entirely within the German dental care settings (3, 15, 17). One broader economic evaluation assessed AI across dermatology, dentistry, and ophthalmology. In that study, the dental component also used a German setting and reported tooth-retention years as the dental outcome (16).

Although all studies addressed caries-related AI applications, the AI approaches were not identical. Schwendicke et al. (17) evaluated a U-Net fully convolutional neural network for proximal caries detection (17). Schwendicke et al. (3) examined how increasing the amount of training data influenced the AI performance and value in caries detection (3). The randomized trial by Schwendicke et al. (15) assessed an AI-supported software application, dentalXrai Pro 1.0.4, in a trial setting (15). Gomez Rossi et al. (16) adapted existing Markov models to compare AI with standard care across three specialties, including dentistry (16).

Despite these differences, all studies used long-term economic modelling, primarily Markov simulation, to estimate downstream consequences of detection and treatment decisions. A further similarity across studies was the use of tooth retention years as the principal effectiveness outcome in the dental analyses. This creates reasonable comparability across studies, even though the source data and modelling assumptions differed.

### 3.2 Diagnostic findings across the included studies

The diagnostic findings consistently showed that AI improved caries detection performance. However, the magnitude and clinical implications of this improvement differed across studies. The clearest direct comparison was reported by Schwendicke et al. (17), where the U-Net model achieved an accuracy of 0.80, compared with a mean accuracy of 0.71 for dentists. In the same study, AI showed higher sensitivity (0.75 vs. 0.36), while specificity was slightly lower but not significantly different from that of dentists (0.83 vs. 0.91). This study therefore provides the main evidence supporting the statement that AI outperformed dentists in diagnostic accuracy (17).

In contrast, Schwendicke et al. (3) did not present a single head-to-head comparison between one AI model and dentists. Instead, that study assessed how enlarging the training dataset altered performance and economic value. The authors found that AI sensitivity and specificity improved nonlinearly as the amount of training data increased, with the largest gain occurring when the training dataset increased from 10% to 25%. Thus, this study contributes evidence that AI performance is influenced not only by model architecture but also by the scale of training data (3).

The randomized trial by Schwendicke et al. (15) found that AI-supported detection was significantly more sensitive than conventional detection. However, in contrast to modelling studies, the higher sensitivity in the AI arm was associated with a greater frequency of invasive treatment decisions. This distinction is important, as it indicates that improved detection does not necessarily translate into better economic outcomes if it leads to less favorable treatment pathways (15). The broader evaluation by Gomez Rossi et al. (16) supported the general proposition that AI as a decision-support system can improve outcomes, but it emphasized that benefits were modest and highly use–case–specific. In the dental component, AI was associated with improved tooth-retention outcomes, but the authors stressed that cost-effectiveness remained sensitive to the fee paid for AI and to the treatment path assumed after diagnosis (16).

Overall, across the four studies, the shared finding is that AI tends to improve detection performance, whereas the main difference lies in whether that improvement is translated into clinically appropriate and economically efficient treatment decisions.

### 3.3 Comparative cost-effectiveness findings

Three of the four included studies favored AI-assisted detection over conventional assessment in the base-case analysis, although the degree of benefit varied. In Schwendicke et al. (3) on value of data and information, AI was associated with greater tooth retention and lower costs than assessment without AI, with mean tooth retention of 62.8 vs. 60.4 years and costs of €378 vs. €419**,** respectively. However, the authors also noted considerable uncertainty and showed that the population's risk profile was more economically influential than further precision around AI cost or accuracy (3).

Similarly, Gomez Rossi et al. (16) reported a favorable dental economic profile for AI, with costs of €320 for AI vs. €342 for control and tooth retention of 62.4 vs. 60.9 years, respectively. However, the gains were described as limited, and the authors emphasized that dominance in favor of AI depended on relatively small differences in the payment assigned to the AI service and in the treatment assumed after diagnosis. This study therefore supports AI’s economic potential, but in a more cautious manner than the dedicated caries modelling papers (16).

The strongest favorable result was reported by Schwendicke et al. (17), in which AI-assisted proximal caries detection was more effective and less costly than dentist assessment without AI. In the base-case analysis, AI achieved 64 vs. 62 tooth-retention years at €298 vs. €322, yielding an ICER of −€13.9 per year, and more than 77% of simulations favored AI. Importantly, the authors stated that this advantage depended on non-restorative management of detected early lesions, meaning that the economic benefit of AI was conditional on conservative downstream care (17). In contrast, the randomized trial by Schwendicke et al. (15) did not demonstrate a clear economic advantage for AI in the base-case analysis. Although AI-supported assessment improved sensitivity, lesions in the AI arm were treated more invasively, and this offset the expected gains. As a result, the AI and control groups had identical effectiveness in terms of tooth retention (49 years) and nearly identical costs (€330 vs. €330). Only 41% of simulations favored AI, compared with 43% favoring no AI, and the authors concluded that cost-effectiveness remained uncertain regardless of the willingness-to-pay threshold (15).

Taken together, the four studies (3, 15–17) suggest a consistent directional signal that AI may improve caries-related economic outcomes, but they also show that this benefit is conditional rather than universal. The three model-based analyses generally favored AI, especially when improved detection translated into non-invasive or micro-invasive management of early lesions (3, 16, 17). In contrast, the trial-based study showed that the expected economic gains may disappear if AI prompts more invasive treatment decisions (15). Therefore, the main similarity across studies is that AI tends to improve diagnostic sensitivity, while the main difference is: whether this diagnostic gain is translated into conservative, value-preserving treatment pathways or into more invasive care with limited economic benefits.

### 3.4 Quality assessment and risk of bias

**Figure 2:**
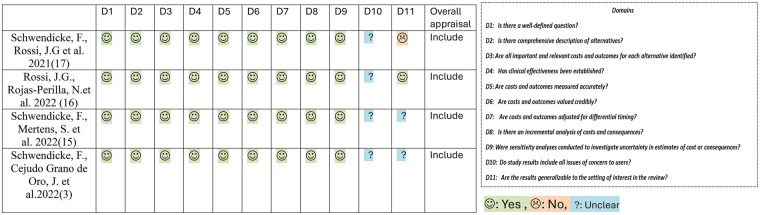
Risk of bias assessment plot.

Details of the bias assessment for each study are presented in Figure 2. All studies clearly defined their research questions and compared relevant alternative approaches. They also identified and included all important costs and outcomes. Clinical effectiveness was well established, and both costs and outcomes were appropriately measured and valued. Adjustments for timing, including discontinuation of costs and outcomes, were clearly reported to ensure temporal accuracy. Incremental cost analyses were performed, and sensitivity analyses addressed key uncertainties. Overall, the risk of bias assessment showed that the included studies had low risk of bias and good methodological reliability (3, 15–17).

In addition to the JBI-based appraisal, the reporting quality of the included economic evaluations was assessed using the CHEERS 2022 checklist (Supplementary Table 2). Four studies explicitly reported following the CHEERS guidelines. Overall, reporting was satisfactory across most key domains, including study objectives, comparators, perspective, time horizon, outcomes, resource use and costs, model structure, uncertainty analysis, and interpretation of findings. However, some important gaps were identified, particularly in reporting health economic analysis plans, heterogeneity, distributional effects, and stakeholder engagement. The discount rate was only partially reported across studies, and the source of funding was not reported in one study. Overall, the findings suggest generally good reporting quality, although several CHEERS 2022 items were incompletely addressed.

## 4 Discussion

This systematic review identified four studies that evaluated the economic implications of AI-assisted screening in oral diseases. All included studies focused on dental or proximal caries detected on bitewing radiographs. As discussed earlier, across studies AI generally improved the diagnostic performance, particularly sensitivity. Three model-based analyses suggested that AI was associated with lower costs and better tooth-retention outcomes than conventional assessment. However, the randomized trial showed that improved sensitivity did not necessarily translate into better cost-effectiveness when it was accompanied by more invasive treatment decisions. These findings indicate that the economic value of AI in dentistry is promising, but it depends strongly on how improved detection is translated into clinical management (3, 15–17).

To the best of our knowledge, this is the first systematic review to synthesize evidence on the economic aspects of AI-assisted oral disease screening. This represents a key contribution of the present study. The findings suggest that AI does not create value through detection alone. Its cost-effectiveness depends on whether earlier or more sensitive detection leads to appropriate downstream treatment decisions. This is particularly important for early lesions, which may be managed non-restoratively. This explains the contrast between the three model-based studies, which generally favored AI, and the randomized trial, where increased invasive treatment offset expected economic gains (3, 15–17). Therefore, the economic impact of AI in dentistry should be evaluated in relation to diagnostic accuracy, treatment pathways, clinical decision-making, and long-term care consequences.

This review also indicates that the economic value of AI is closely linked to the quality and scale of the underlying training data. In one included study (3), enlarging the dataset used for AI training improved sensitivity and specificity and was associated with improved cost-effectiveness, although the incremental benefit diminished as the dataset became larger. This finding reinforces the importance of representative, high-quality, and externally validated datasets for developing clinically reliable and economically meaningful AI applications in dentistry. Recent guidance from the American Dental Association similarly emphasizes the importance of independent validation datasets and careful evaluation of dental image-analysis systems before broader clinical adoption (21).

Although the findings are encouraging, the current evidence base remains limited to caries-related AI applications and is largely derived from model-based evaluations. This narrow evidence base restricts the extent to which broader conclusions can be drawn about the economic value of AI across oral healthcare. This is particularly relevant given the substantial global burden of oral diseases, which affect billions of people worldwide and require scalable, context-sensitive, and economically sustainable solutions (22).

Overall, this review suggests that AI-assisted caries detection may improve both clinical and economic outcomes, but its value is conditional rather than automatic. The most consistent message across studies is not simply that AI is more accurate, but that its economic value depends on the interaction between diagnostic gain, treatment decisions, implementation context, and data quality. Strengthening the evidence base will therefore require rigorously designed studies that combine robust AI validation with equally robust economic evaluation.

### 4.1 Limitations and future research

This review has several limitations that should be considered when interpreting its findings. All included dental economic evaluations were conducted in the German healthcare setting, which limits generalizability to other healthcare systems with different reimbursement structures, workforce costs, resource availability, and oral healthcare delivery models. In addition, only four studies met the inclusion criteria, which restricted the scope for synthesis and precluded quantitative meta-analysis. Heterogeneity in study design, modelling assumptions, analytic perspectives, and effectiveness outcomes, including tooth-retention years and QALYs, may also have affected comparability across studies. Furthermore, restricting the review to English-language, peer-reviewed studies may have excluded relevant evidence from grey literature, health technology assessment reports, and real-world implementation studies.

These limitations also highlight important priorities for future research. First, economic evaluations of AI in dentistry should extend beyond caries detection to other high-burden conditions, such as oral cancer and periodontal disease, where no eligible evidence was identified. Second, future studies should adopt prospective, real-world, and multicentre designs that assess not only diagnostic performance but also downstream treatment decisions, workflow impact, implementation costs, and reimbursement mechanisms. Third, greater use of standardized reporting frameworks, such as CHEERS 2022 for health economic evaluations and CONSORT-AI for prospective AI intervention trials, would improve transparency, reproducibility, and comparability across studies (12, 23). Fourth, the development and validation of AI systems should rely on larger, more representative, and independently validated datasets to enhance robustness and reduce bias. More context-specific economic evaluations are also needed in low- and middle-income countries, where disease burden, cost structures, and access to care differ from high-income settings.

In addition, Future research should also address broader implementation challenges, including data sharing, privacy protection, algorithmic bias, regulatory preparedness, and the costs of maintaining AI-enabled systems in routine dental practice. Addressing these issues will require collaboration among clinicians, economists, AI developers, regulators, and health system decision-makers. Such efforts are essential to generate policy-relevant evidence and to support equitable and context-sensitive integration of AI into oral healthcare.

## 5 Conclusion

This systematic review suggests that AI-assisted screening, particularly for dental caries, may improve diagnostic performance and support longer tooth retention at comparable or lower long-term costs when integrated with appropriate clinical management. To the best of our knowledge, this is the first systematic review to synthesize economic evidence on AI-assisted screening for oral diseases. The findings have important implications for clinical practice, policy, and research. For clinicians, AI may support earlier and more consistent lesion detection. For policymakers and payers, the evidence may inform reimbursement and implementation decisions. For researchers and developers, the findings highlight the need for stronger external validation, closer alignment between AI outputs and treatment pathways, and context-specific economic evaluations. AI-assisted screening may also be valuable in remote or underserved settings where access to specialist diagnostic support is limited. Future studies should prioritize multicentre, real-world economic evaluations across diverse oral conditions and healthcare systems to support equitable and evidence-based integration of AI into oral healthcare.

## Data Availability

All data analyzed in this study are included in this published article and its Supplementary Material. No new datasets were generated during the current study.

## Supplementary material

Supplementary Table 1

Database-specific search strategies.

SUPPLEMENTARY TABLE 2

CHEERS 2022 checklist-based reporting assessment of the included studies.

## References

[1] Hamet P Tremblay J. Artificial intelligence in medicine. Metab Clin Exp. (2017) 69s:S36–40. 10.1016/j.metabol.2017.01.011 28126242

[2] Khanagar SB Al-Ehaideb A Maganur PC Vishwanathaiah S Patil S Baeshen HA. Developments, application, and performance of artificial intelligence in dentistry - A systematic review. J Dent Sci. (2021) 16(1):508–22. 10.1016/j.jds.2020.06.019 33384840 PMC7770297

[3] Schwendicke F Cejudo Grano de Oro J Garcia Cantu A Meyer-Lueckel H Chaurasia A Krois J. Artificial intelligence for caries detection: value of data and information. J Dent Res. (2022) 101(11):1350–6. 10.1177/00220345221113756 35996332 PMC9516598

[4] Chen YW Stanley K Att W. Artificial intelligence in dentistry: current applications and future perspectives. Quintessence Int. (2020) 51(3):248–57. 10.3290/j.qi.a43952 32020135

[5] Hinton G. Deep learning-A technology with the potential to transform health care. Jama. (2018) 320(11):1101–2. 10.1001/jama.2018.11100 30178065

[6] Miotto R Wang F Wang S Jiang X Dudley JT. Deep learning for healthcare: review, opportunities and challenges. Brief Bioinform. (2018) 19(6):1236–46. 10.1093/bib/bbx044 28481991 PMC6455466

[7] Bonny T Al Nassan W Obaideen K Al Mallahi MN Mohammad Y El-Damanhoury HM. Contemporary role and applications of artificial intelligence in dentistry. F1000Res. (2023) 12:1179. 10.12688/f1000research.140204.1 37942018 PMC10630586

[8] Ding H Wu J Zhao W Matinlinna JP Burrow MF Tsoi JKH. Artificial intelligence in dentistry-A review. Front Dent Med. (2023) 4:1085251. 10.3389/fdmed.2023.1085251 39935549 PMC11811754

[9] He J Baxter SL Xu J Xu J Zhou X Zhang K. The practical implementation of artificial intelligence technologies in medicine. Nat Med. (2019) 25(1):30–6. 10.1038/s41591-018-0307-0 30617336 PMC6995276

[10] Bharadwaj C. AI in dentistry: benefits, applications and real-world cases (2025). Available online at: https://appinventiv.com/blog/ai-in-dentistry/ (Accessed July 13, 2025)

[11] Kastrup N Holst-Kristensen A Valentin J. Landscape and challenges in economic evaluations of artificial intelligence in healthcare: a systematic review of methodology. BMC Digit Health. (2024):2:1–12. 10.1186/s44247-024-00088-7

[12] Husereau D Drummond M Augustovski F de Bekker-Grob E Briggs AH Carswell C. Consolidated health economic evaluation reporting standards 2022 (CHEERS 2022) statement: updated reporting guidance for health economic evaluations. Value Health. (2022) 25(1):3–9. 10.1016/j.jval.2021.11.1351 35031096

[13] Robinson R. Cost-effectiveness analysis. Br Med J. (1993) 307(6907):793–5. 10.1136/bmj.307.6907.793 8219957 PMC1696433

[14] Rudmik L Drummond M. Health economic evaluation: important principles and methodology. Laryngoscope. (2013) 123(6):1341–7. 10.1002/lary.23943 23483522

[15] Schwendicke F Mertens S Cantu AG Chaurasia A Meyer-Lueckel H Krois J. Cost-effectiveness of AI for caries detection: randomized trial. J Dent. (2022) 119:104080. 10.1016/j.jdent.2022.104080 35245626

[16] Gomez Rossi J Rojas-Perilla N Krois J Schwendicke F. Cost-effectiveness of artificial intelligence as a decision-support system applied to the detection and grading of melanoma, dental caries, and diabetic retinopathy. JAMA Netw Open. (2022) 5(3):e220269. 10.1001/jamanetworkopen.2022.0269 35289862 PMC8924723

[17] Schwendicke F Rossi JG Göstemeyer G Elhennawy K Cantu AG Gaudin R. Cost-effectiveness of artificial intelligence for proximal caries detection. J Dent Res. (2021) 100(4):369–76. 10.1177/0022034520972335 33198554 PMC7985854

[18] Hettiarachchi RM Kularatna S Downes MJ Byrnes J Kroon J Lalloo R. The cost-effectiveness of oral health interventions: a systematic review of cost-utility analyses. Community Dent Oral Epidemiol. (2018) 46(2):118–24. 10.1111/cdoe.12336 28925508

[19] Page M McKenzie J Bossuyt P Boutron I Hoffmann T Mulrow C. The PRISMA 2020 statement: an updated guideline for reporting systematic reviews. Br Med J. (2021) 372:n71. 10.1136/bmj.n71 33782057 PMC8005924

[20] Gomersall JS Jadotte YT Xue Y Lockwood S Riddle D Preda A. Conducting systematic reviews of economic evaluations. Int J Evid Based Healthc. (2015) 13(3):170–8. 10.1097/XEB.0000000000000063 26288063

[21] American Dental Association. Dentistry—evaluation of Dental Image Analysis Systems Using Augmented/artificial Intelligence. ADA Technical Report No. 1109. Chicago: American Dental Association (2024/2025).

[22] World Health Organization. Global oral health status report: towards universal health coverage for oral health by 2030. Geneva: WHO (2022). Available online at: WHO website (Accessed April 8, 2025)

[23] Liu X Cruz Rivera S Moher D Calvert MJ Denniston AK Ashrafian H. Reporting guidelines for clinical trial reports for interventions involving artificial intelligence: the CONSORT-AI extension. Lancet Digit Health. (2020) 2(10):e537–48. 10.1016/S2589-7500(20)30218-1 33328048 PMC8183333

