# Supplementary material for: Economic evaluation of AI-based oral disease screening: a systematic review
Source: Front Med Technol. 2026 Jun 25;8:1792401. doi: 10.3389/fmedt.2026.1792401 (PMC13346071; doi:10.3389/fmedt.2026.1792401)
Supplement: Supplementary file 1 [file Table1.docx]

**Supplementary Table -1**

**Search Terms:**

| 1. **PubMed (Total Studies: - 11)** |
| --- |
| ("artificial intelligence*"[MeSH Terms] OR "artificial intelligence"[Title/Abstract] OR "machine learning"[Title/Abstract] OR "deep learning"[Title/Abstract] OR "AI"[Title/Abstract] OR "ML"[Title/Abstract] OR "DL"[Title/Abstract] OR "neural networks"[Title/Abstract] OR "NLP"[Title/Abstract] OR "image processing"[Title/Abstract] OR "computer vision"[Title/Abstract] OR "convolutional neural"[Title/Abstract] OR "deep feature learning"[Title/Abstract] OR "artificial neural networks"[Title/Abstract] OR "pattern recognition automated"[Title/Abstract]) AND ("oral health"[MeSH Terms] OR "oral cavity"[Title/Abstract] OR "oral diseases"[Title/Abstract] OR "oral cancer"[Title/Abstract] OR "oral lesions"[Title/Abstract] OR "oral health"[Title/Abstract] OR "oral hygiene"[Title/Abstract] OR "oral mucosa"[Title/Abstract] OR "oral pathology"[Title/Abstract] OR "Dental"[Title/Abstract]) AND ("cost benefit analysis"[MeSH Terms] OR "cost effectiveness analysis"[Title/Abstract] OR "economic evaluation"[Title/Abstract] OR "cost utility analysis"[Title/Abstract] OR "marginal analysis"[Title/Abstract] OR "cost benefit data"[Title/Abstract] OR ("health care economics"[Title/Abstract] AND "Organizations"[Title/Abstract]) OR "cost analysis"[Title/Abstract] OR "economic impact"[Title/Abstract] OR "economic modelling"[Title/Abstract]) |
| 1. **EMBASE (Total Studies : - 180)** |
| ('artificial intelligence*'/exp OR 'artificial intelligence':ti,ab OR 'machine learning':ti,ab OR 'deep learning':ti,ab OR AI:ti,ab OR ML:ti,ab OR DL:ti,ab OR 'neural networks':ti,ab OR NLP:ti,ab OR 'image processing':ti,ab OR 'computer vision':ti,ab OR 'convolutional neural':ti,ab OR 'deep feature learning':ti,ab OR 'artificial neural networks':ti,ab OR 'pattern recognition automated':ti,ab) AND ('oral health'/exp OR 'oral cavity':ti,ab OR 'oral diseases':ti,ab OR 'oral cancer':ti,ab OR 'oral lesions':ti,ab OR 'oral health':ti,ab OR 'oral hygiene':ti,ab OR 'oral mucosa':ti,ab OR 'oral pathology':ti,ab OR Dental:ti,ab) AND ('cost benefit analysis'/exp OR 'cost effectiveness analysis':ti,ab OR 'economic evaluation':ti,ab OR 'cost utility analysis':ti,ab OR 'marginal analysis':ti,ab OR 'cost benefit data':ti,ab OR ('health care economics':ti,ab AND Organizations:ti,ab) OR 'cost analysis':ti,ab OR 'economic impact':ti,ab OR 'economic modelling':ti,ab) |
| 1. **EBSCO – CINAHL (Total Studies – 5)** |
| ((MH "artificial intelligence*+") OR (TI "artificial intelligence" OR AB "artificial intelligence") OR (TI "machine learning" OR AB "machine learning") OR (TI "deep learning" OR AB "deep learning") OR (TI AI OR AB AI) OR (TI ML OR AB ML) OR (TI DL OR AB DL) OR (TI "neural networks" OR AB "neural networks") OR (TI NLP OR AB NLP) OR (TI "image processing" OR AB "image processing") OR (TI "computer vision" OR AB "computer vision") OR (TI "convolutional neural" OR AB "convolutional neural") OR (TI "deep feature learning" OR AB "deep feature learning") OR (TI "artificial neural networks" OR AB "artificial neural networks") OR (TI "pattern recognition automated" OR AB "pattern recognition automated")) AND ((MH "oral health+") OR (TI "oral cavity" OR AB "oral cavity") OR (TI "oral diseases" OR AB "oral diseases") OR (TI "oral cancer" OR AB "oral cancer") OR (TI "oral lesions" OR AB "oral lesions") OR (TI "oral health" OR AB "oral health") OR (TI "oral hygiene" OR AB "oral hygiene") OR (TI "oral mucosa" OR AB "oral mucosa") OR (TI "oral pathology" OR AB "oral pathology") OR (TI Dental OR AB Dental)) AND ((MH "cost benefit analysis+") OR (TI "cost effectiveness analysis" OR AB "cost effectiveness analysis") OR (TI "economic evaluation" OR AB "economic evaluation") OR (TI "cost utility analysis" OR AB "cost utility analysis") OR (TI "marginal analysis" OR AB "marginal analysis") OR (TI "cost benefit data" OR AB "cost benefit data") OR ((TI "health care economics" OR AB "health care economics") AND (TI Organizations OR AB Organizations)) OR (TI "cost analysis" OR AB "cost analysis") OR (TI **"**economic impact" OR AB "economic impact") OR (TI "economic modelling" OR AB "economic modelling")) |
| 1. **Scopus (Total Studies: 79)** |
| ("artificial intelligence*" OR "artificial intelligence" OR "machine learning" OR "deep learning" OR AI OR ML OR DL OR "neural networks" OR NLP OR "image processing" OR "computer vision" OR "convolutional neural" OR "deep feature learning" OR "artificial neural networks" OR "pattern recognition automated") AND ("oral health" OR "oral cavity" OR "oral diseases" OR "oral cancer" OR "oral lesions" OR "oral health" OR "oral hygiene" OR "oral mucosa" OR "oral pathology" OR Dental) AND ("cost benefit analysis" OR "cost effectiveness analysis" OR "economic evaluation" OR "cost utility analysis" OR "marginal analysis" OR "cost benefit data" OR ("health care economics" AND Organizations) OR "cost analysis" OR "economic impact" OR "economic modelling") |
| 1. **Cochrane Library (Total Studies: 20)** |
| ("artificial intelligence" OR "machine learning" OR "deep learning" OR AI OR ML OR DL OR "neural networks" OR NLP OR "image processing" OR "computer vision" OR "convolutional neural" OR "deep feature learning" OR "artificial neural networks" OR "pattern recognition automated") AND ("oral health" OR "oral cavity" OR "oral diseases" OR "oral cancer" OR "oral lesions" OR "oral health" OR "oral hygiene" OR "oral mucosa" OR "oral pathology" OR Dental) AND ("cost benefit analysis" OR "cost effectiveness analysis" OR "economic evaluation" OR "cost utility analysis" OR "marginal analysis" OR "cost benefit data" OR "health care economics" OR "cost analysis" OR "economic impact" OR "economic modelling") in Title Abstract Keyword - (Word variations have been searched) |
| 1. **Web of Science (Total No of studies: 39)** |
| ("artificial intelligence*" OR "artificial intelligence" OR "machine learning" OR "deep learning" OR AI OR ML OR DL OR "neural networks" OR NLP OR "image processing" OR "computer vision" OR "convolutional neural" OR "deep feature learning" OR "artificial neural networks" OR "pattern recognition automated") AND ("oral health" OR "oral cavity" OR "oral diseases" OR "oral cancer" OR "oral lesions" OR "oral health" OR "oral hygiene" OR "oral mucosa" OR "oral pathology" OR Dental) AND ("cost benefit analysis" OR "cost effectiveness analysis" OR "economic evaluation" OR "cost utility analysis" OR "marginal analysis" OR "cost benefit data" OR ("health care economics" AND Organizations) OR "cost analysis" OR "economic impact" OR "economic modelling") |

Supplementary Table 2. CHEERS 2022 reporting assessment of included studies

| **CHEERS 2022** | **Schwendicke et al., 2022 [3]** | **Schwendicke et al., 2022 [15]** | **Gomez Rossi et al., 2022[16]** | **Schwendicke et al., 2021 [17]** |
| --- | --- | --- | --- | --- |
| 1. Title | R | R | R | R |
| 2. Abstract | R | R | R | R |
| 3. Background and objectives | R | R | R | R |
| 4. Health economic analysis plan | NR | NR | NR | NR |
| 5. Study population | R | R | R | R |
| 6. Setting and location | R | R | R | R |
| 7. Comparators | R | R | R | R |
| 8. Perspective | R | R | R | R |
| 9. Time horizon | R | R | R | R |
| 10. Discount rate | PR | PR | PR | PR |
| 11. Selection of outcomes | R | R | R | R |
| 12. Measurement of outcomes | R | R | R | R |
| 13. Valuation of outcomes | NA | NA | R | NA |
| 14. Measurement and valuation of resources and costs | R | R | R | R |
| 15. Currency, price date, and conversion | R | R | R | R |
| 16. Rationale and description of model | R | R | R | R |
| 17. Analytics and assumptions | R | R | R | R |
| 18. Characterising heterogeneity | NR | NR | NR | NR |
| 19. Characterising distributional effects | NR | NR | NR | NR |
| 20. Characterising uncertainty | R | R | R | R |
| 21. Approach to engagement with patients and others affected by the study | NR | NR | NR | NR |
| 22. Study parameters | R | R | R | R |
| 23. Summary of main results | R | R | R | R |
| 24. Effect of uncertainty | R | R | R | R |
| 25. Effect of engagement with patients and others affected by the study | NR | NR | NR | NR |
| 26. Study findings, limitations, generalisability, and current knowledge | R | R | R | R |
| 27. Source of funding | R | NR | R | R |
| 28. Conflicts of interest | R | R | R | R |

**Abbreviations:** **R = Reported; PR = Partially reported (not in sufficient detail); NR = Not reported; NA = Not applicable.**
